# Haemodynamics in a patient with Fontan physiology undergoing laparoscopic cholecystectomy

**DOI:** 10.1007/s12471-015-0704-7

**Published:** 2015-06-02

**Authors:** S.J.A. Pans, R.R.J. van Kimmenade, J.P. Ruurda, F.J. Meijboom, G.T. Sieswerda, B. van Zaane

**Affiliations:** 1 Department of Anesthesiology, University Medical Center Utrecht, PO Box 85500, 3508 GA Utrecht, The Netherlands; 2 Department of Cardiology, Maastricht University Medical Center, Maastricht, The Netherlands; 3 Department of Surgery, University Medical Center Utrecht, Utrecht, The Netherlands; 4 Department of Cardiology, University Medical Center Utrecht, Utrecht, The Netherlands

**Keywords:** Congenital heart disease, Univentricular heart, Fontan physiology, Laparoscopic surgery, Cardiac output

## Abstract

Laparoscopic surgery in patients with Fontan circulation is a haemodynamic challenge; venous return may be compromised by insufflation of carbon dioxide into the abdomen (increasing intra-abdominal pressure), the use of reverse Trendelenburg position and positive pressure ventilation. Combined with an increase in pulmonary vascular resistance due to hypercarbia, cardiac output may be reduced. However, for non-haemodynamic reasons, laparoscopic surgery has advantages over open surgery: less postoperative pain, shorter hospital stay, a reduction in postoperative wound infections and a reduction of respiratory complications. In this case report, we present a patient with Fontan circulation who underwent uneventful laparoscopic cholecystectomy.

In a monoventricle circulation, such as the Fontan circulation, the venous return is connected directly to the pulmonary circulation without interposition of a right ventricle. In this situation, surgery in general, but laparoscopic surgery in particular, is a haemodynamic challenge. Venous return may be compromised by insufflation of carbon dioxide into the abdomen (increasing intra-abdominal pressure (IAP)), the use of reverse Trendelenburg position and positive pressure ventilation.[1]. Combined with an increase in pulmonary vascular resistance (PVR) due to hypercarbia, cardiac output may be reduced [2, 3]. However, for non-haemodynamic reasons, laparoscopic surgery has advantages over open surgery: less postoperative pain, shorter hospital stay, a reduction of postoperative wound infections and a reduction of respiratory complications [4–6]. In the literature, not much information is found describing the haemodynamics in Fontan patients undergoing laparoscopic surgery. However, in this case report, we present a patient with Fontan circulation who underwent uneventful laparoscopic cholecystectomy.

A 23-year-old man presented for elective cholecystectomy for cholecystitis. He had a history of a tricuspid atresia with a hypoplastic right ventricle, and a ventricular septal defect. He had a normal relation of great arteries. He was palliated with a total cavopulmonary Fontan: bidirectional Glenn anastomosis and an intracardiac tunnel with fenestration, to lead the venous return from inferior caval vein and hepatic veins to the pulmonary artery (Fig. 1b). His preoperative cardiac evaluation showed an adequate Fontan circulation with good ventricular function. After starting standard monitoring (ECG, SaO_2_), a peripheral intravenous line and a radial arterial line, the patient was pre-hydrated with 1 l of Ringer’s lactate to optimise pre-load, which is important as we explain later. Anaesthesia was induced and maintained with standard dosages of propofol, remifentanil and rocuronium. The trachea was intubated, and a central venous line was inserted in the right jugular vein. Transoesophageal echocardiography and a cardiac output meter were used to monitor cardiac function.

**Fig. 1:**
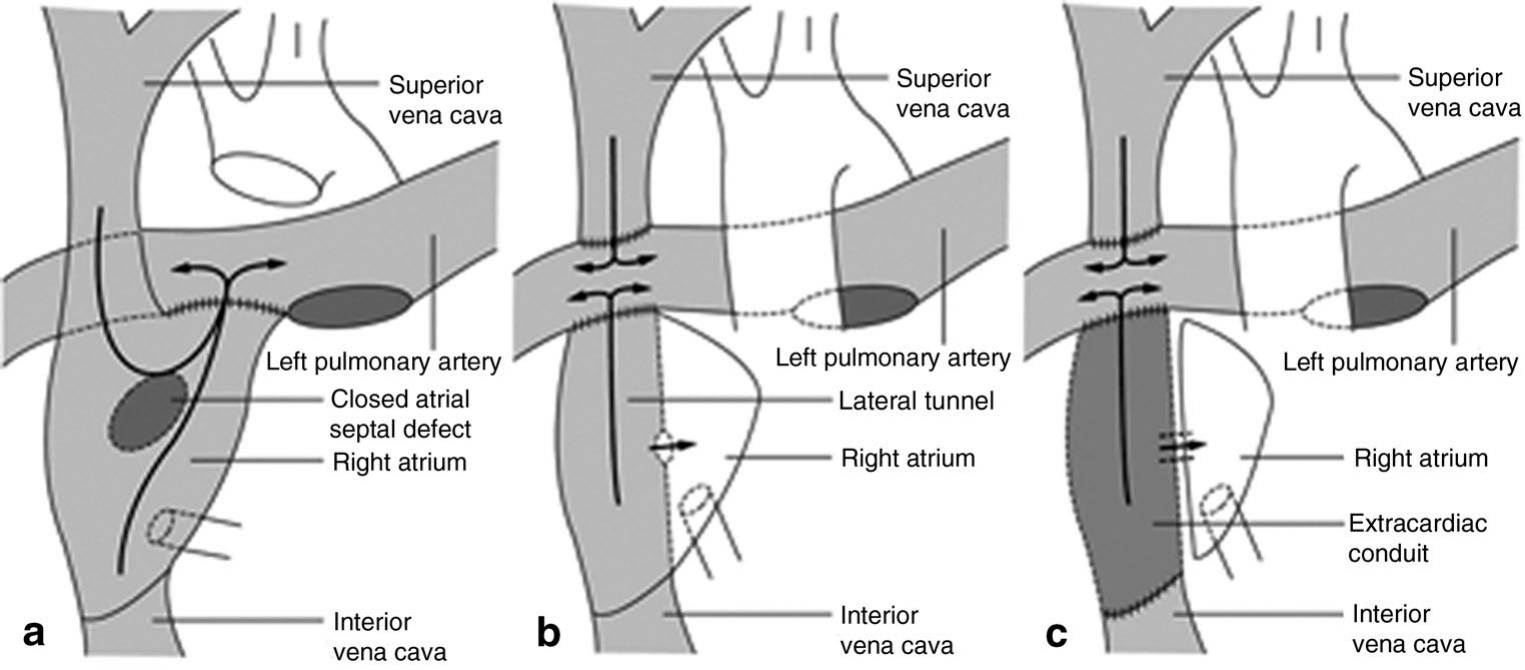
Different types of Fontan circulation. **a** Atriopulmonary connection. **b** Intracardiac total cavopulmonary connection (lateral tunnel). Situation of the patient described in this case report. **c** Extracardiac total cavopulmonary connection. Reprinted by permission from Macmillan Publishers Ltd. [11]

Prior to insufflation of carbon dioxide, he was ventilated with a peak inspiratory pressure of 26 cmH_2_O and a positive expiratory pressure of 2 cmH_2_O. At the start of the procedure, the blood pressure was 100/40 mmHg; the cardiac output, 4.0 l/min; and central venous pressure (CVP), 11 mmHg (Fig. 2, start of procedure)**.** After creating the pneumoperitoneum, with a maximal IAP of 10 mmHg, systolic blood pressure increased to 150/70 mmHg, cardiac output increased to a maximum of 7.8 l/min and the CVP increased to 20 mmHg (Fig. 2, insufflation)**.** Ventilation was adjusted to keep end-tidal CO_2_ at 4.0 kPa. To maintain pre-load, 2000 ml of Ringer’s lactate was given during the procedure.

**Fig. 2:**
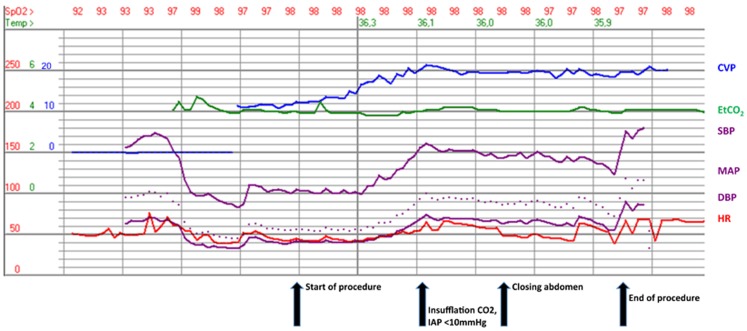
Tracing of haemodynamic and ventilatory parameters (*CVP* central venous pressure, *EtCO*_*2*_ end-tidal carbon dioxide, *SBP* systolic blood pressure, *MAP* mean arterial pressure, *DBP* diastolic blood pressure, *HR* heart rate)

Transoesophageal echocardiography showed a good function of the systemic ventricle before and during pneumoperitoneum. During the procedure, the blood pressure was stable at 150/70 mmHg, cardiac output decreased to 5.8 l/min and the CVP was 20 mmHg. After the uneventful surgical procedure, the patient emerged from the anaesthesia and was extubated. He was transferred to the intensive care unit for postoperative care and on postoperative day 2 discharged home.

In a normal cardiovascular system, the pulmonary and systemic circulations are connected in series, powered by a bi-ventricular heart. The primary function of the right ventricle is to supply the lungs with blood, and to supply the left side of the heart with enough pre-load to produce adequate cardiac output. In an uncorrected monoventricular malformation, such as tricuspid atresia and right/left ventricle hypoplasia, the pulmonary and systemic circulation are connected in parallel. This leads to chronic arterial desaturation, and congestive heart failure due to the continuous overload of the single ventricle [6]. To palliate these monoventricular malformations, patients undergo several procedures to create a Fontan circulation in which the pulmonary and systemic circulation are separated again. The systemic venous return is connected directly to the pulmonary circulation, without interposition of a ventricle (Fig. 1). Consequently, varying conditions in the systemic venous circulation are directly translated in the pulmonary blood flow. Adequate cardiac output in Fontan circulation depends on pre-load, pulmonary vascular resistance, atrioventricular valve function, cardiac rhythm and ventricular function [7]. In a normal circulation, a modest elevation of PVR—for example, due to hypercarbia—can be overcome by the right ventricle: pulmonary blood flow can be maintained without increase of CVP. This is not possible in the Fontan circulation; pulmonary blood flow will decrease, unless compensated by an increased CVP [8].

The classical view is that in laparoscopic surgery, the increase in IAP causes a reduction in pre-load by impaired venous return and an increase in afterload [9]. In Fontan circulation, this would mean a decreased cardiac output, and thus laparoscopic surgery would be contraindicated [2, 10]. However, more recent data show that IAPs up to 12 mmHg have limited effects on these parameters, and may even increase cardiac output [1, 10]. In a study on cardiac output in children undergoing laparoscopy with low IAP (5 mmHg), an increase in cardiac output was observed when the IAP was lower than the CVP. The theory is that blood recruited from splanchnic capacity vessels creates a net increased venous return and an increase in cardiac output. This only results in compression of the inferior vena cava, with a decrease in venous return and cardiac output, if the IAP rises above the CVP [1, 10].

As patients with a Fontan circulation have a chronically increased CVP, it is likely that a moderate increase in IAP, that is, 10–12 mmHg, increases venous return, the driving force of the Fontan circulation and the cardiac output. This is consistent with the observation in our patient.

The main concern during any surgical procedure in a patient with Fontan circulation is to maintain adequate venous return, which determines pulmonary flow and cardiac output. This case report shows that laparoscopic surgery is feasible in patients with a Fontan circulation when IAP is kept below the CVP, that is, below 10–12 mmHg and systemic venous pressure is maintained—or elevated—with substantial pre-hydration and fluid administration during the procedure. Continuous monitoring of CVP during the procedure is mandatory.

In conclusion, in this case report, we show that laparoscopic approach to cholecystectomy in patients with Fontan circulation is not contraindicated when the IAP is kept below the CVP, and venous return to the lungs is adequate.

## Funding

None.

## Conflict of interests

None declared.
